# Supplementary material for: Proteomic analysis reveals key differences between squamous cell carcinomas and adenocarcinomas across multiple tissues
Source: Nat Commun. 2022 Jul 18;13:4167. doi: 10.1038/s41467-022-31719-0 (PMC9293992; doi:10.1038/s41467-022-31719-0)
Supplement: Supplementary file 1 — Supplementary Information [file 41467_2022_31719_MOESM1_ESM.pdf]

Supplementary Fig.1

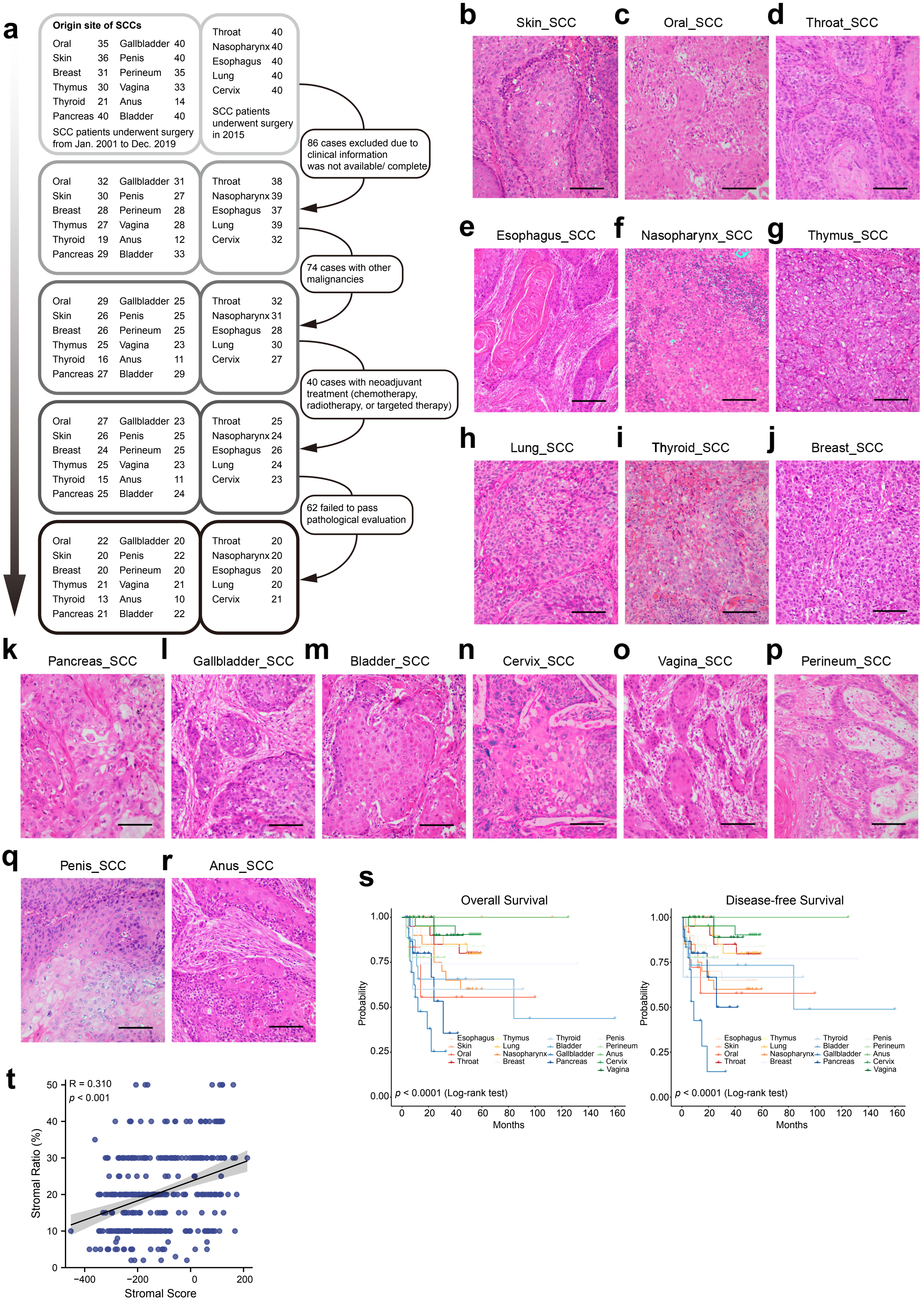

Supplementary Fig. 1 Representative morphology and prognosis of pan-SCC cohort. **a** The quality control and sample filtering standards of sample collection in this cohort. Among the 262 excluded patients, 86 patients were with no/complete standard clinical information (not including TNM stage information etc. for 12 SCCs with a lower incidence), 74 patients with other malignancies, 40 cases with neoadjuvant treatment, and 62 patients failed to pass pathological evaluation. Supplementary Table 1a shows the surgery year distribution of all 333 SCC patients. **b-r** Representative HE staining of 17 SCCs. Scale bar, 100 μm. **s** Kaplan-Meier plots show the overall and disease-free survival of 274 patients of 17 SCCs (*p* value from log-rank test). **t** A scatterplot showed the correlation between Stromal Score (x axis) and Stromal Ratio (y axis). Pairwise Spearman correlation. Source data are provided as a Source Data file.

Supplementary Fig. 2

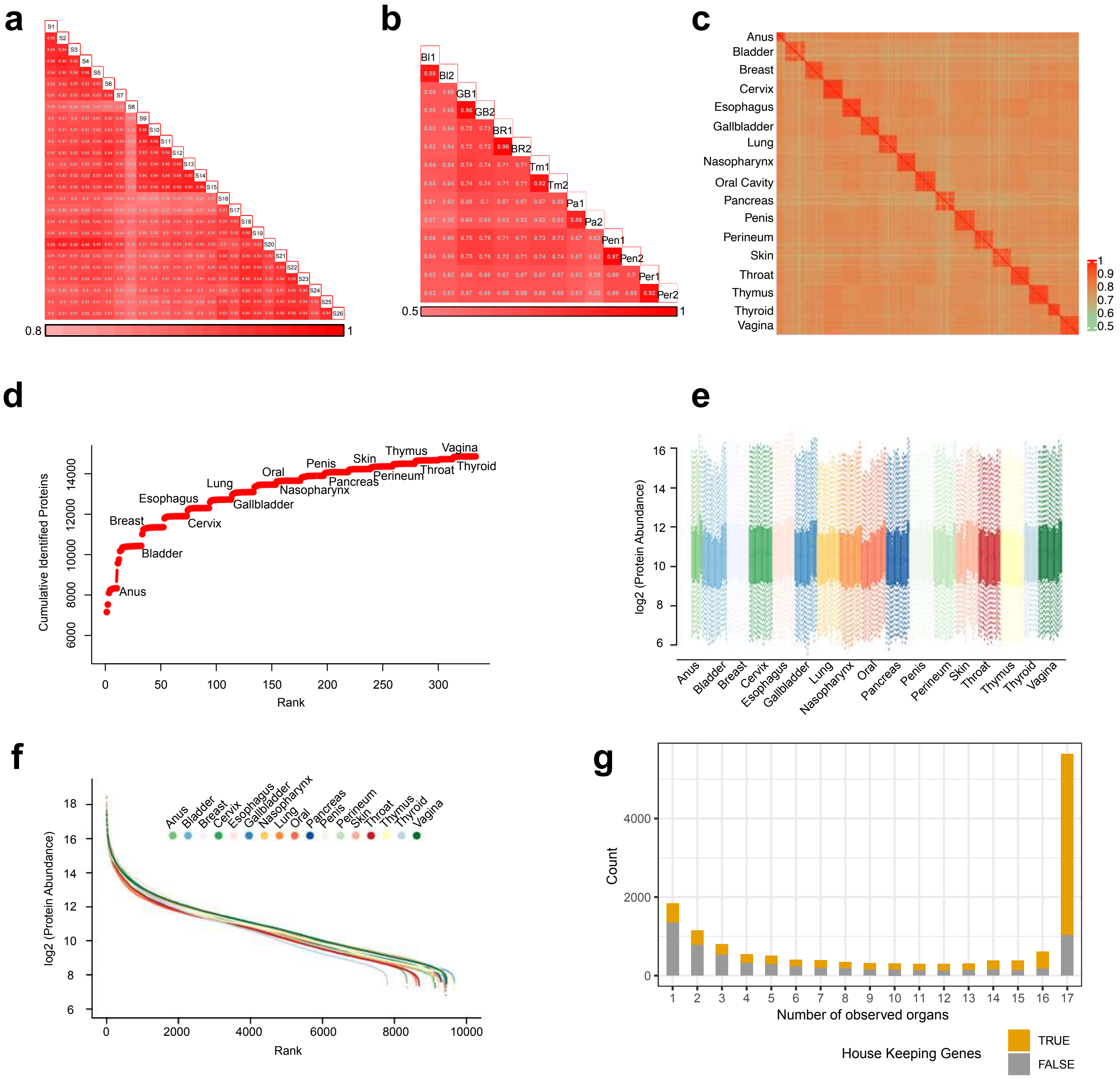

h

Dataset	Counts	Description
Pro1	14,840	Protein groups identified against Uniprot homo sapiens database (downloaded in July, 2019)
Pro2	14,598	Union set of protein groups identified in at least 1/3 samples in at least one SCC
Pro3	5,130	Intersection of protein groups expressed in at least 1/2 samples in at least one SCC
Pro4	6,213	Union set of intersection of protein groups expressed in at least 1/2 samples in Common SCCs and Rare SCCs (Separation between Common and Rare SCCs)
Pro5	1,500	1,500 top mad in union set of 20% bottom mad of 17 individual SCCs (Hierarchical clustering SCC subtype classifier building)

Supplementary Fig. 2 Quality assessments for MS data.

a Longitudinal quality control of mass spectrometry using tryptic digest of HEK293T cells by representing the pairwise Spearman's correlation coefficients of the samples. **b** Spearman's correlation coefficients for replicate proteome profiling of 2 bladder, 2 gallbladder, 2 breast, 2 thymus, 2 pancreatic, 2 penis, and 2 perineum SCCs. Repeat experiments with the same samples have good reproducibility, with a high level of correlation (average, 0.92; range, 0.86-0.97). **c** Spearman's correlation coefficients for all 333 MS runs. The median correlation coefficient among these samples was 0.74, and the maximum and minimum values were 0.99 and 0.56, respectively. **d** Cumulative number of protein identifications across 17 SCCs. **e**, **f** Overview of the proteomics profile of pan-SCC patients. Distribution of log2-transformed protein abundance of identified proteins in 333 proteome samples across 17 SCCs that passed quality control. **g** Distribution of the number of proteins quantified across different numbers of SCCs. A total of 5,648 proteins were presented in all 17 SCCs, and 4,618 proteins were classified as HK proteins among them. **h** Summary of proteomic datasets at different levels. 14,598 proteins were quantified in at least 1/3 samples in at least one SCC.

Supplementary Fig.3

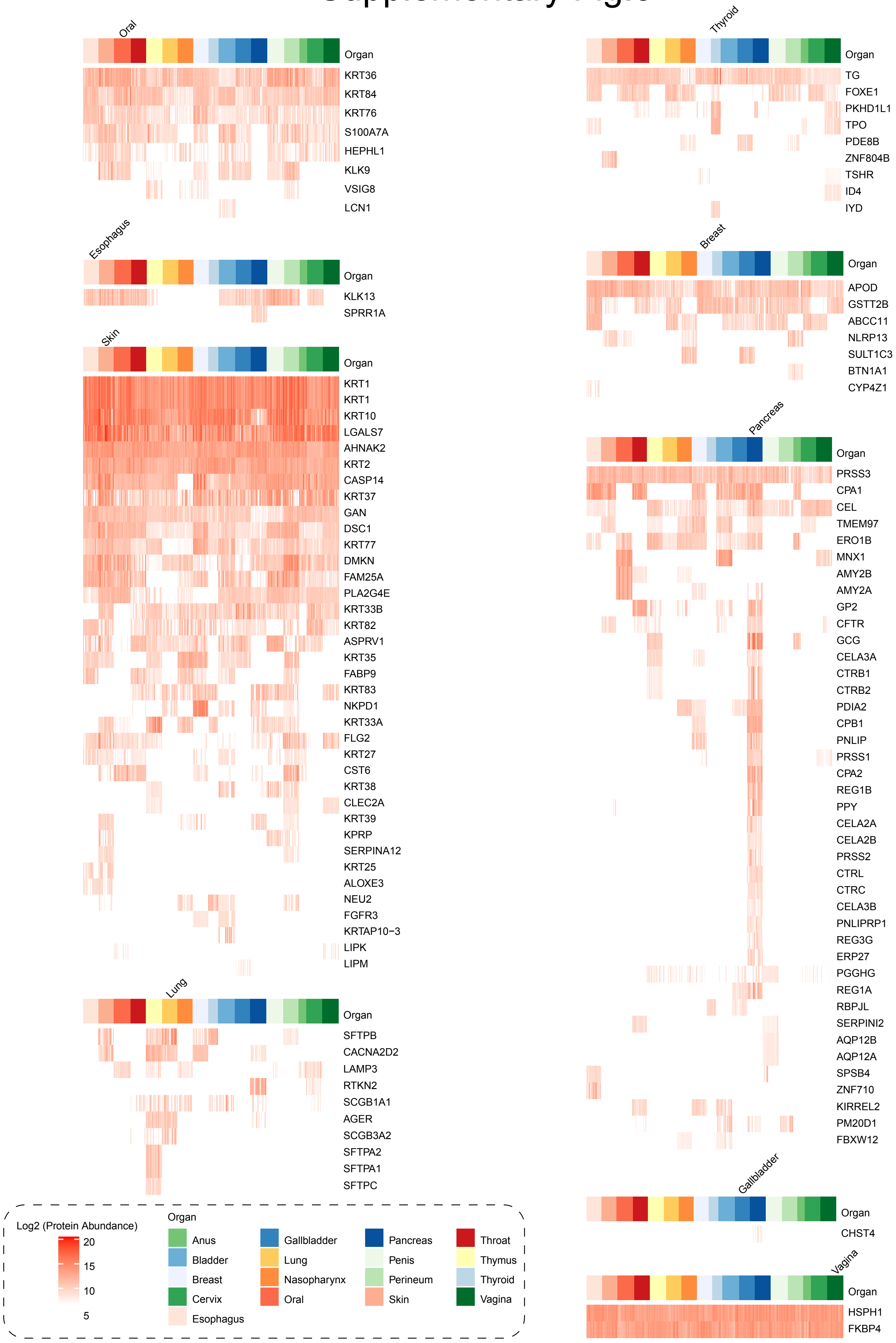

Supplementary Fig. 3 The tissue signature protein (Human protein atlas) expression pattern across pan-SCC cohort.

A total of 9 lists of signature proteins that were loss of specific expression in certain organ SCCs, including oral, esophagus, skin, lung, thyroid, breast, pancreas, gallbladder, and vagina. Log2-transformed protein abundance was shown.

Supplementary Fig.4

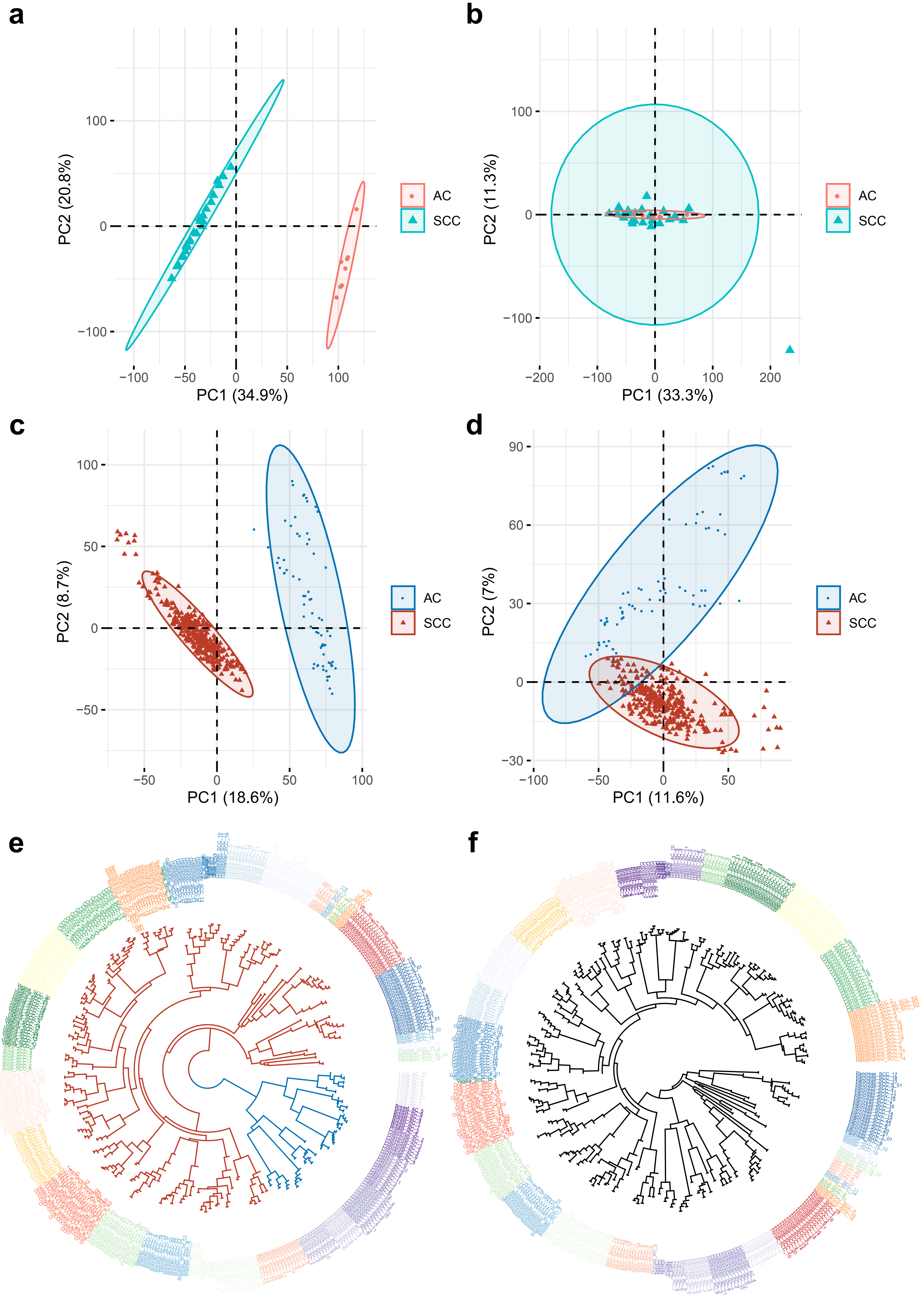

Supplementary Fig. 4 Batch effect correction for pan-SCC and pan-AC cohort.

a, b Principal-component analysis of quality control of mass spectrometry using tryptic digest of HEK293T cells before and after batch effect removal of pan-SCC cohort and pan-AC cohort. **c, d** Principal-component analysis of pan-SCC cohort and pan-AC cohort before and after batch effect removal. **e, f** Dendrogram of the hierarchical relationship before and after batch effect removal based on pairwise Euclidean distance from Ward's method.

Supplementary Fig.5

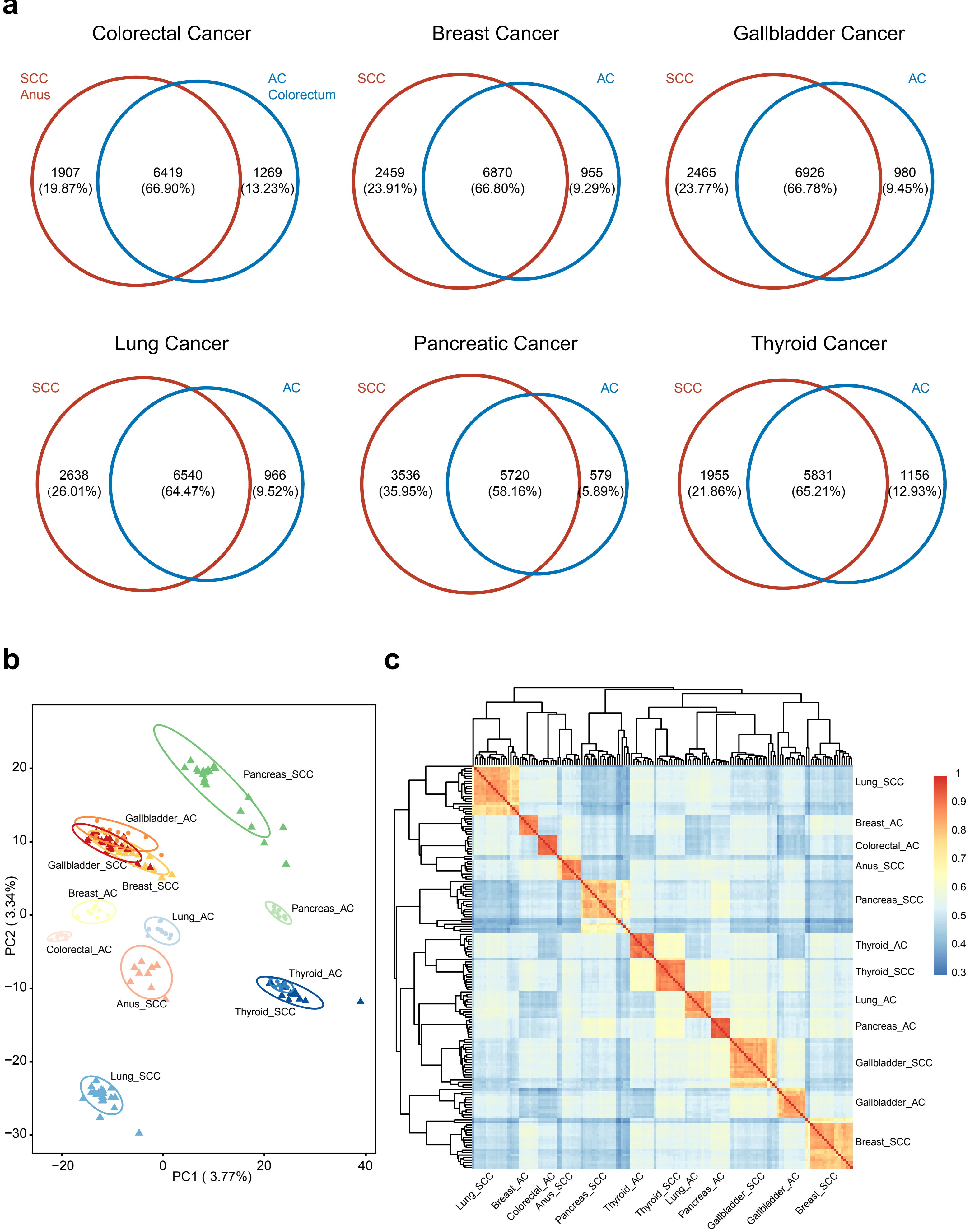

Supplementary Fig. 5 Pairwise comparison between 6 pairs of SCCs and ACs.

a Venn diagrams showing comparison of identified proteins using SCCs versus ACs originating from the same organ, including colorectum (anus in pan-SCC cohort), breast, gallbladder, lung, pancreas, and thyroid. **b** Principal-component analysis of 6 pairs of SCCs and ACs showing diverse distance between ACs and SCCs from the same organ. **c** Correlation matrix of 6 pairs of AC and SCC samples (Spearman's correlation coefficients) showing the same trend as the principal-component analysis.

Supplementary Fig.6

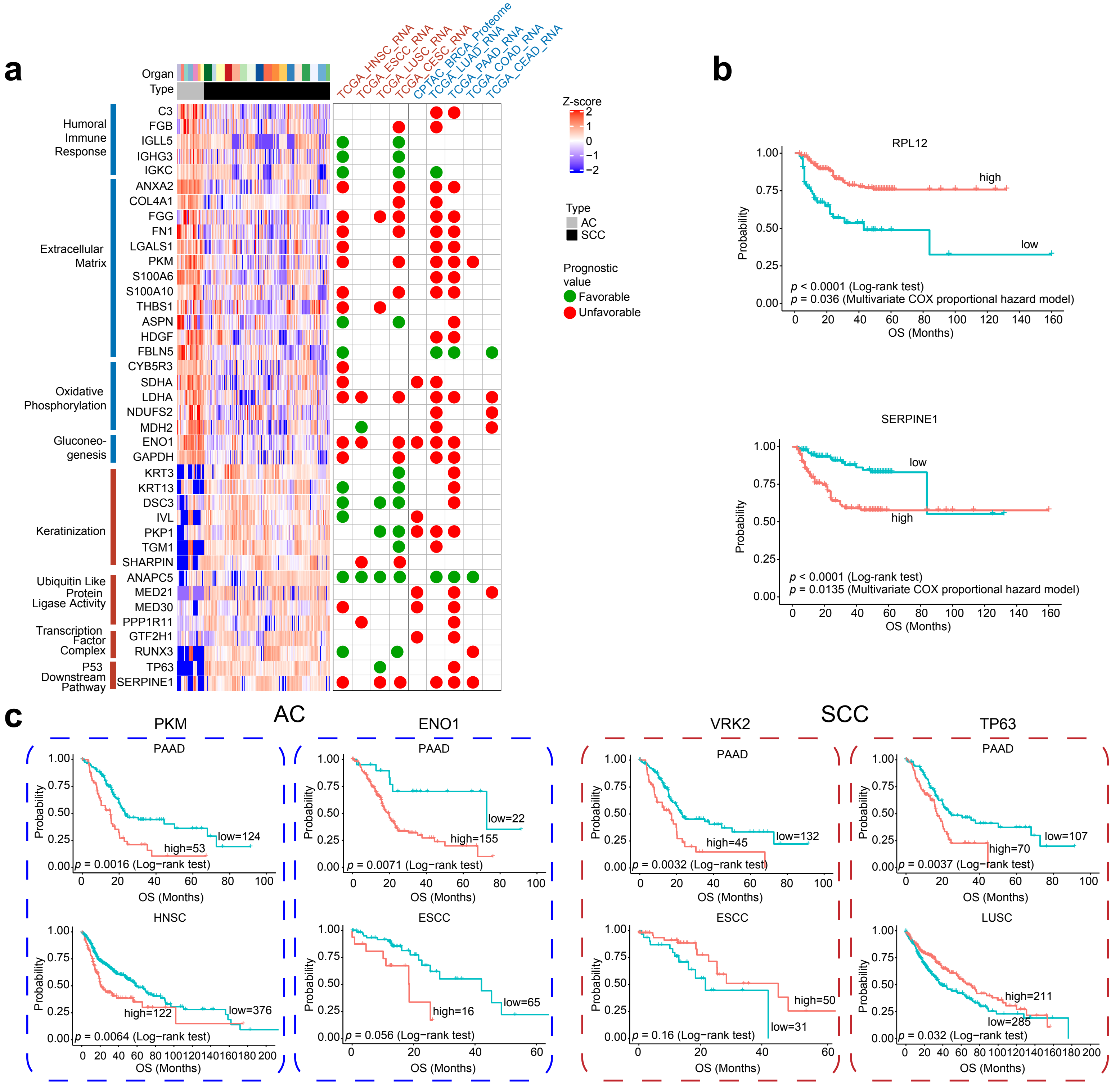

Supplementary Fig. 6 Prognostic values of DEPs in pan-ACs or pan-SCCs enriched pathways.

a The prognostic value of differentially expressed proteins in ACs enriched pathways (humoral immune response, extracellular matrix, oxidative phosphorylation, and gluconeogenesis) and SCC enriched pathway (keratinization, ubiquitin like protein ligase activity, transcription factor complex, and p53 downstream pathway) by exploring pan-SCC cohort (this study) and 9 TCGA datasets. *P* values of pan-SCC cohort were from multivariate COX proportional hazard model (including protein expression, organ, and histology), and *p* values for 9 TCGA datasets were from log-rank test. **b** Kaplan-Meier survival curves for RPL12 and SERPINE1 with *p* value from multivariate COX proportional hazard model (including protein expression, age, gender, histology, organ, and stage) labeled. **c** Representative kinases and TFs from regulation networks of pan-ACs and pan-SCCs and their association with prognosis (*p* value from log-rank test).

Supplementary Fig.7

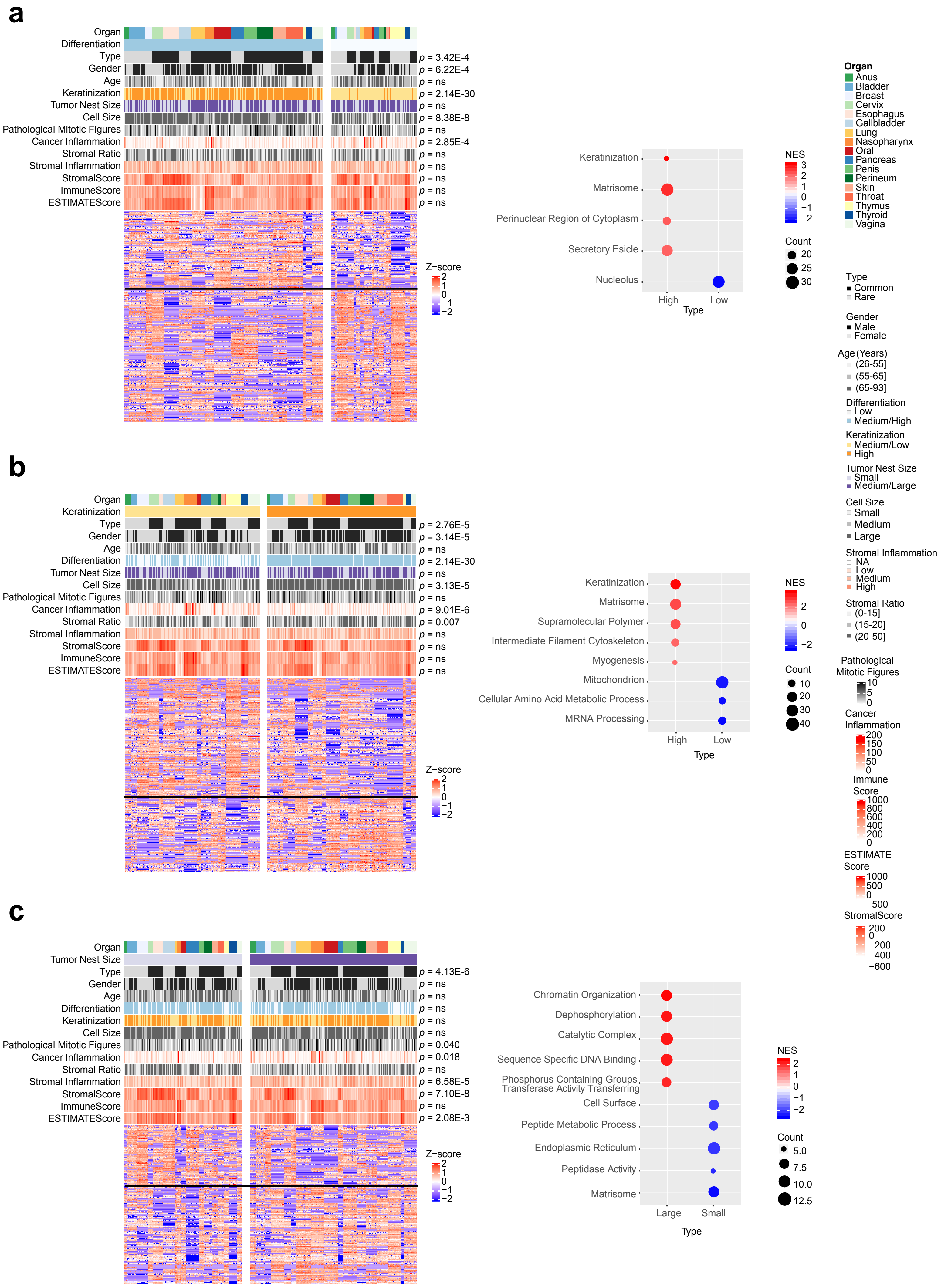

Supplementary Fig. 7 Clinicopathological comparison of proteomics for pan-SCC cohort.

The association of (**a**) differentiation status, (**b**) keratinization status, and (**c**) cell nest size with 13 variables (two-sided Fisher's exact test was used for categorical variables and two-sided Wilcoxon rank-sum test was used for continuous variables), and the heatmap and enriched pathways of significantly DEPs (Wilcoxon rank-sum test, BH $p < 0.05$, fold change > 2) in tumors with high/medium or low differentiation, high or medium/low keratinization, and large/medium or small cell nest size. NES, normalized enrichment score.

Supplementary Fig.8

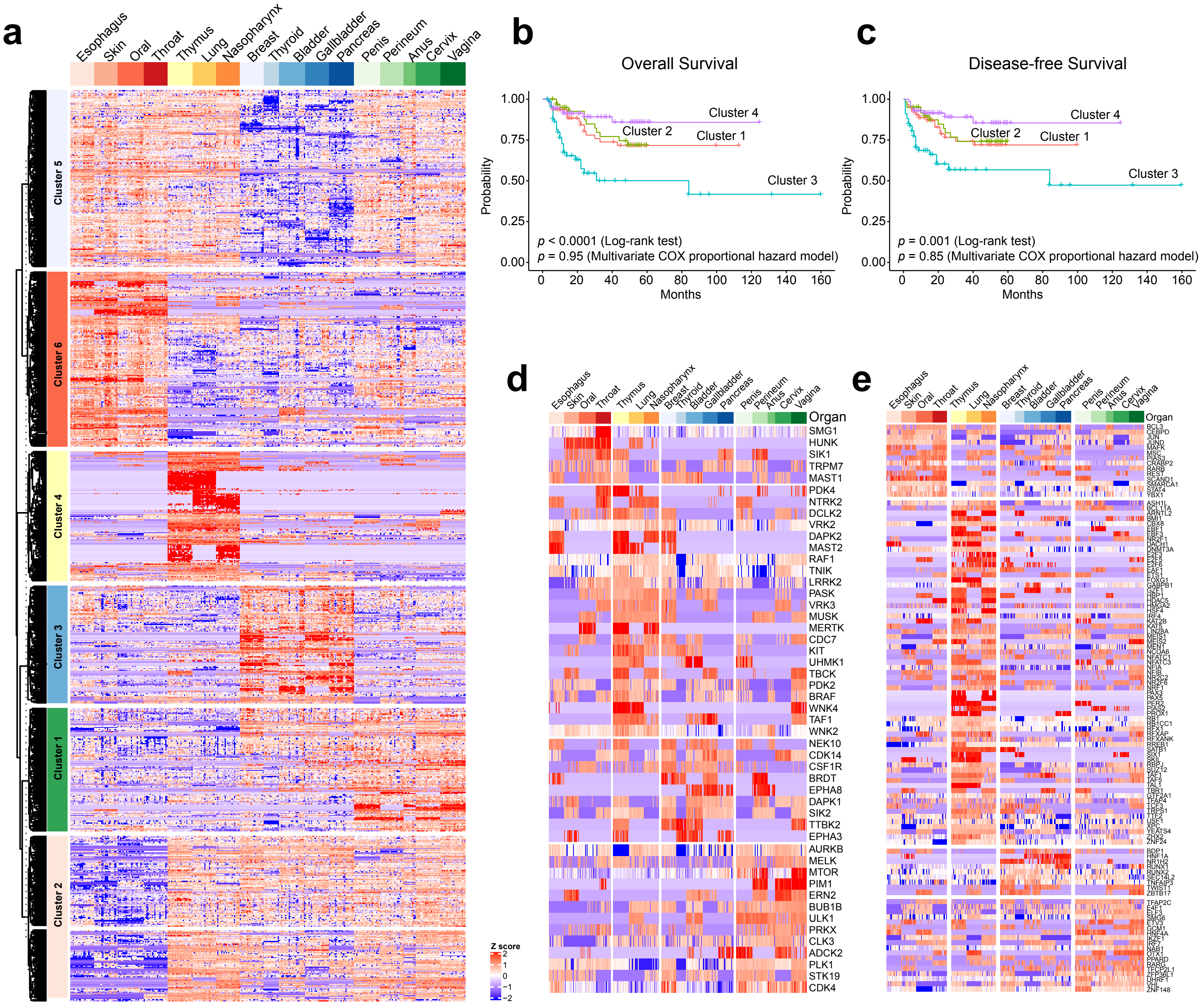

Supplementary Fig. 8 The heatmaps of differential expressed proteins, kinases, and TFs for proteomic clusters with differential prognosis.

a A heatmap showed the DEPs of a Kruskal-Wallis test comparing the four proteomic clusters ($p < 0.05$). **b, c** Kaplan-Meier plots show the overall and disease-free survival of the four proteomic clusters (p value from log-rank test, and multivariate COX proportional hazard model including subtypes, age, gender, stage, histology, and organs). **d** A heatmap showed the differential expressed kinases of a Kruskal-Wallis test comparing the four proteomic clusters ($p < 0.05$). **e** A heatmap showed the differential expressed TFs of a Kruskal-Wallis test comparing the four proteomic clusters ($p < 0.05$).

Supplementary Fig.9

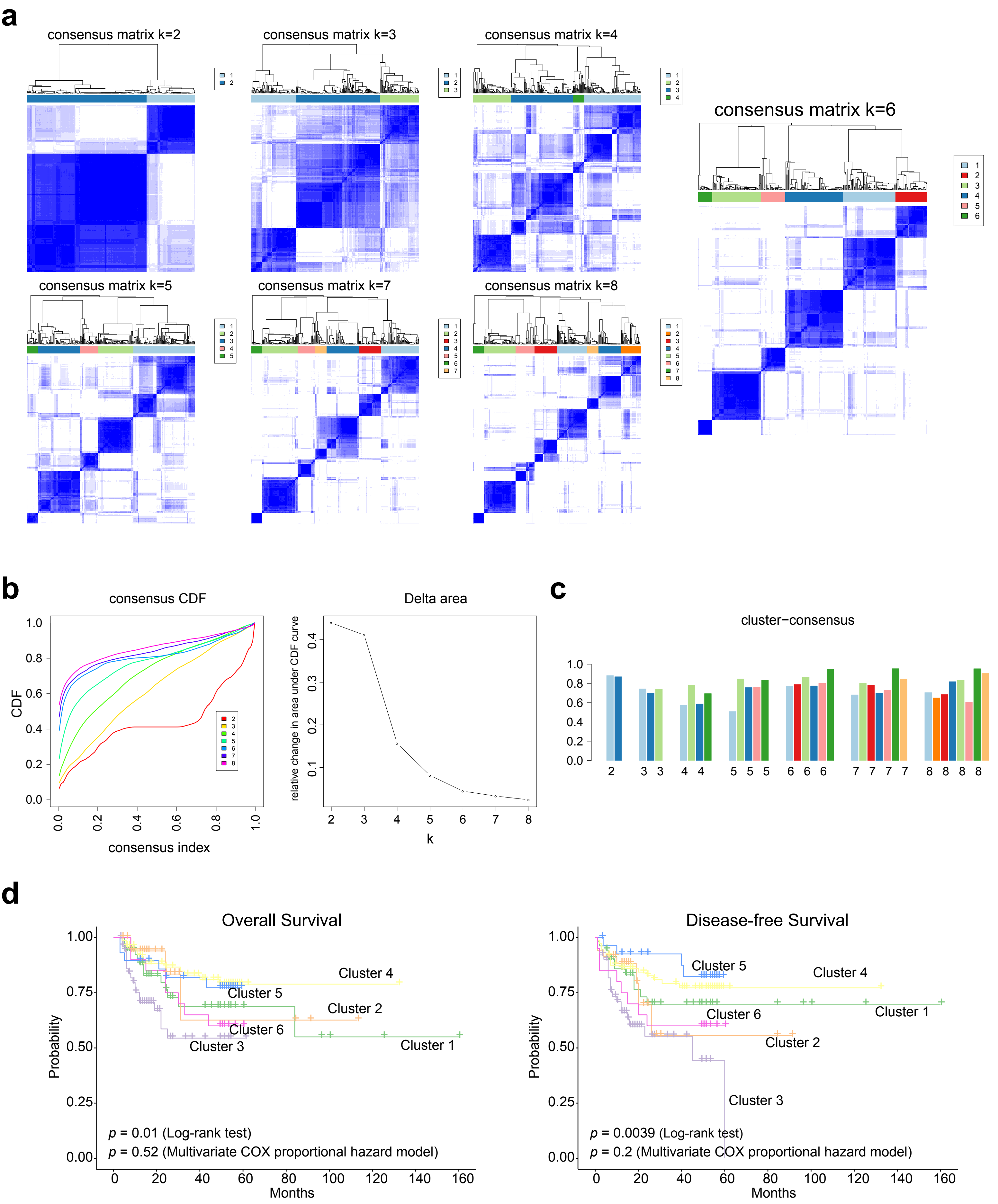

Supplementary Fig. 9 Consensus clustering based on microenvironmental cell gene signatures identified five immune clusters.

a Subgroups were identified based on xCell score of pan-SCC cohort (n=333) by consensus clustering. k was tested from 2 to 8 and consensus clustering was based on 1,000 resampled datasets. We consider k=6 to be the preferred solution and use this to arrange the samples shown in Fig. 5. **b** Consensus cumulative distribution function (CDF) plot, delta area (change in CDF area) plot, and (**c**) cluster-consensus were shown. **d** Kaplan-Meier plots show the overall and disease-free survival of the six subtypes (*p* value from log-rank test, and multivariate COX proportional hazard model including subtypes, age, gender, stage, histology, and organs).

Supplementary Fig.10

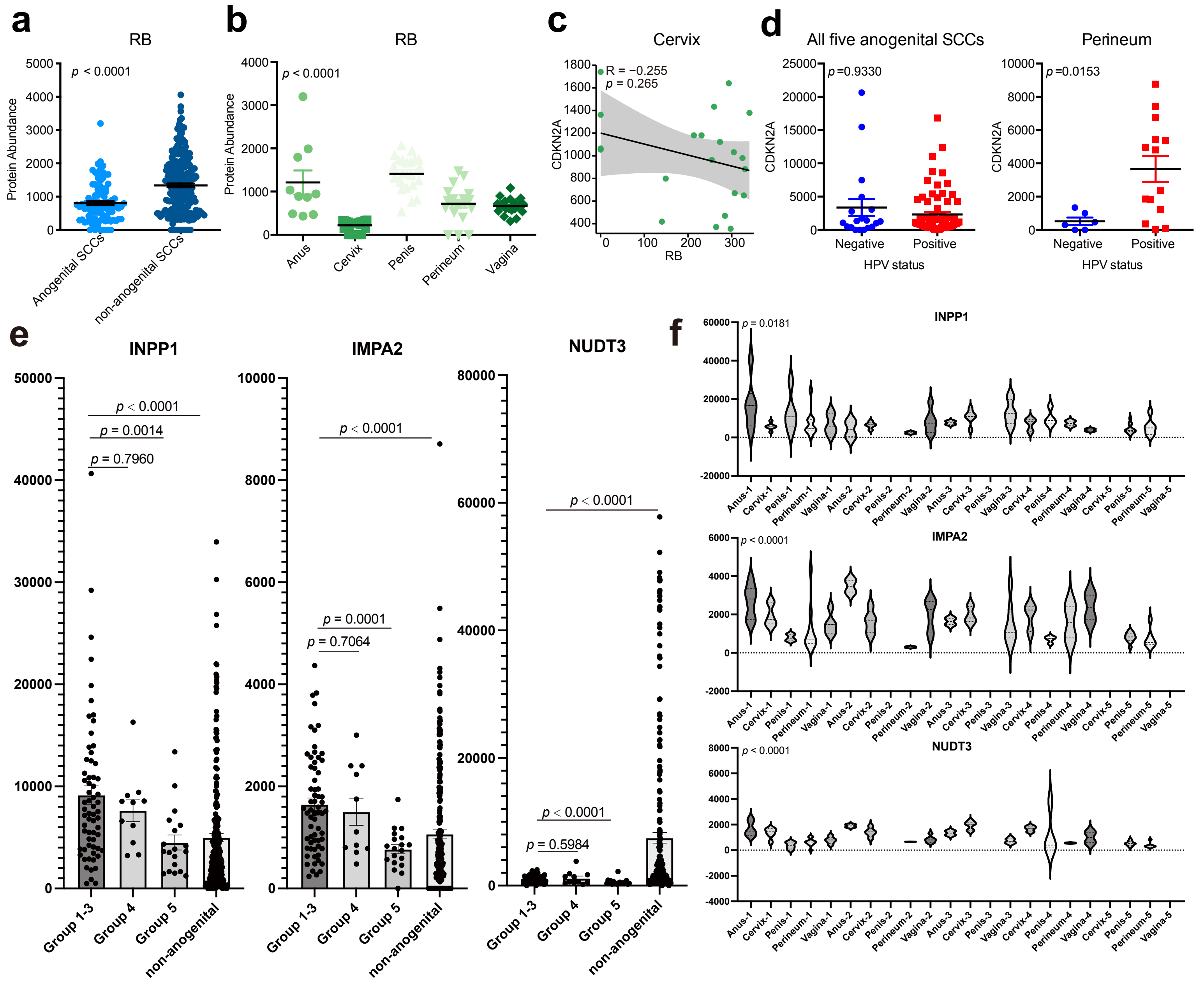

Supplementary Fig. 10 HPV related protein expression in anogenital SCCs.

a RB expression in anogenital SCCs and non-anogenital SCCs, Wilcoxon rank-sum test. $n_{anogenital\ SCCs}$ = 94 and $n_{non\text{-}anogenital\ SCCs}$ = 239 biologically independent samples examined. **b** RB expression in five anogenital SCCs, two-sided Kruskal-Wallis test. n_{anus} = 10, n_{cervix} = 21, n_{penis} = 22, $n_{perineum}$ = 20, and n_{vagina}= 21 biologically independent samples examined. **c** Scatterplots showed the correlation between RB (x axis) and CDKN2A (y axis) in cervical SCCs. Pairwise Spearman correlation. **d** Scatterplots showed the CDKN2A expression comparison between HPV negative and HPV positive patients in all five anogenital SCCs (left) and SCC of perineum (right). Two-sided t test. Left: $n_{negative}$ = 19 and $n_{postive}$ = 75 biologically independent samples examined. Right: $n_{negative}$ = 6 and $n_{postive}$ = 14 biologically independent samples examined. **e** Boxplots showed protein expression of molecules in Inositol phosphate catabolic process (INPP1, IMPA2, and NUDT3) of Group 1-3 (n = 64), Group 4 (n = 11), Group 5 (n = 19), and non-anogenital SCCs (n = 239) (biologically independent samples examined, two-sided Wilcoxon rank-sum test). **f** Violin plots showed protein expression of molecules in Inositol phosphate catabolic process (INPP1, IMPA2, and NUDT3) across 5 groups of 5 anogenital SCCs, Kruskal-Wallis test. All data are expressed as mean values +/- SEM. Source data are provided as a Source Data file.

Supplementary Fig.11

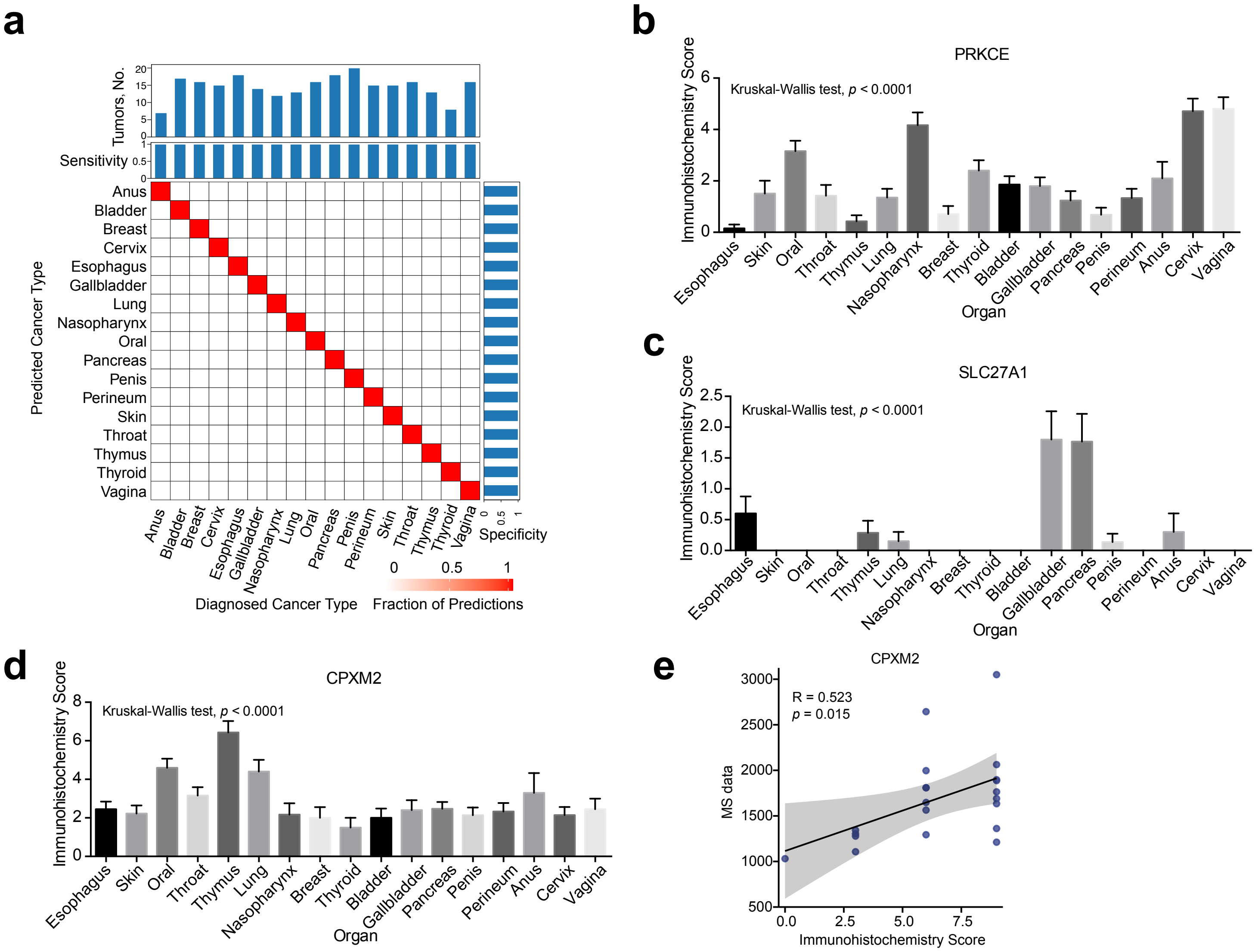

Supplementary Fig. 11 The immunohistochemistry score of three validated markers. **a** Performance of the classifier across 17 SCCs in training set. True (established) cancer types are displayed horizontally and predicted cancer types are displayed vertically. The number of tumors of each cancer type in the cohort is shown at the top, and sensitivity and specificity of the predictions are indicated at the top and right. Boxplots showing the immunohistochemistry score of PRKCE (**b**), SLC27A1 (**c**), and CPXM2 (**d**) in 17 SCCs. two-sided Kruskal-Wallis test. $n_{anus} = 10$, $n_{bladder} = 22$, $n_{breast} = 20$, $n_{cervix} = 21$, $n_{esophagus} = 20$, $n_{gallbladder} = 20$, $n_{lung} = 20$, $n_{nasopharynx} = 20$, $n_{oral} = 22$, $n_{pancreas} = 21$, $n_{penis} = 22$, $n_{perineum} = 20$, $n_{skin} = 20$, $n_{throat} = 20$, $n_{thymus} = 21$, $n_{thyroid} = 13$, and $n_{vagina} = 21$ biologically independent samples examined. **e** Significant correlation between the immunohistochemistry data and proteomics data (p =0.015) of CPXM2 in thymus SCC. Pairwise Spearman correlation. All data are expressed as mean values +/- SEM. Source data are provided as a Source Data file.
