# Supplementary material for: Proteomic analysis reveals key differences between squamous cell carcinomas and adenocarcinomas across multiple tissues
Source: Nat Commun. 2022 Jul 18;13:4167. doi: 10.1038/s41467-022-31719-0 (PMC9293992; doi:10.1038/s41467-022-31719-0)
Supplement: Supplementary file 3 — Description of Additional Supplementary Files [file 41467_2022_31719_MOESM3_ESM.docx]

**Description of Additional Supplementary Files**

**Supplementary Data 1:** Clinical characteristics of pan-SCC patients. Related to Fig. 1.

Year of surgery: The distribution of surgery year of 333 SCC patients.

Clinical characteristics: Clinical characteristics of 333 SCC patients.

Statistics: Clinical characteristics of 333 SCC patients. The associations between SCC types and clinicopathological characteristics was calculated by two-sided Fisher’s exact test for categorical variables and two-sided Wilcoxon rank-sum test for continuous variables for the 333 patients.

Multivariate COX model: Association between clinicopathological characteristics and OS/DFS by multivariate COX proportional hazards model.

Proteome quantification matrix: 14,598 proteins at gene level. This sheet contains information of 14,598 proteins quantified at gene level in all 333 SCC samples.

**Supplementary Data 2:** Proteomic characteristics of SCCs compared with ACs. Related to Fig. 2.

Proteome quantification matrix: 10,414 proteins at gene level. This sheet contains information of 10,414 proteins quantified at gene level in all 69 AC samples.

DEPs: Differentially expressed proteins between SCC (n=333) and AC (n=69) samples. The statistical significance was calculated by two-sided Wilcoxon rank-sum test. P-values were adjusted by the Benjamini-Hochberg (BH) algorithm and significant calls were made based on an BH adjusted p-value < 0.05 and |logFC| ≥1.

Pathways: Significantly altered pathways between SCCs (n=333) and ACs (n=69). Pathway enrichment significance were calculated using one-sided Fisher's exact test following Benjamini-Hochberg adjustment. Pathways with Benjamini-Hochberg adjusted p-value < 0.05 were selected.

Prognostic analysis: The prognostic analysis of a list of differentially expressed proteins of top 10 altered pathways between SCCs and ACs among the pan-SCC cohort and 9 TCGA cohorts.

**Supplementary Data 3:** Proteomic features in rare SCCs compared with common SCCs. Related to Fig. 3.

DEPs: Differentially expressed proteins between Common-SCC(n=206) and Rare-SCC(n=127) samples. The statistical significance was calculated by two-sided Wilcoxon rank-sum test. P-values were corrected by Benjamini-Hochberg (BH) algorithm and significant calls were made based on BH adjusted p-value < 0.05 and |logFC|≥1.

Pathways: Significantly altered pathways between Common-SCC(n=206) and Rare-SCC(n=127). Two-sided Wilcoxon rank-sum test, BH p < 0.05, fold change > 2.

FISH analysis: *PLIN1* fluorescence in situ hybridization results in 170 SCCs.

**Supplementary Data 4:** Proteome based hierarchical clustering of 17 SCCs. Related to Fig. 4.

DEPs: Differentially expressed proteins of 4 proteomic clusters. Kruskal-Wallis test (BH adjusted *p* < 0.05) was used to identify proteins that were differentially expressed between the four clusters.

Pathways: Significantly altered pathways of 4 proteomic clusters.

**Supplementary Data 5:** Immune landscape of SCCs and their potential druggable insights. Related to Fig. 5.

xCell: Stromal and Immune Signatures derived via xCell (http://xcell.ucsf.edu/).

Pathways: Significantly altered pathways of 6 proteomic subtypes.

DEPs: Differentially expressed proteins of 6 immune subtypes. A simple linear model and two-sided moderated t-statistics, implemented with the R/Bioconductor package limma v.3.40.6, were used to identify differentially expressed proteins between the 6 immune subtypes, and the following cut-off criteria were used: (1) all BH-adjusted P values should be less than 0.05 compared to the other subtypes; (2) fold change (expressed as log2(ratio of average protein abundance between immune subtypes) ≥ 1.5 or ≤ -1.5); and (3) at least 50% expression in one subtype. Because the differentially proteins of subtype 6 are overly abundant, we adjusted the filter criteria (fold change (expressed as log2(ratio of average protein abundance between immune subtypes) ≥ 3 or ≤ -3)).

**Supplementary Data 6:** Characterization of HPV-related SCCs. Related to Fig. 6.

HPV infection status: HPV infection status of anogenital SCCs.

HPV prevalence: HPV type-specific prevalence in 5 anogenital SCCs.

p53 expression: p53 expression in HPV positive and negative anogenital SCC patients.

Pathways: Eight patterns of differential pathway regulation between the 5 groups with differential HPV infection.
